# ^1^H NMR Spectroscopy Combined with Machine-Learning Algorithm for Origin Recognition of Chinese Famous Green Tea Longjing Tea

**DOI:** 10.3390/foods13172702

**Published:** 2024-08-27

**Authors:** Zhiwei Hou, Yugu Jin, Zhe Gu, Ran Zhang, Zhucheng Su, Sitong Liu

**Affiliations:** 1 College of Tea Science and Tea Culture, Zhejiang A & F University, 666 Wusu Street, Hangzhou 311300, China; jinyugu0011@163.com (Y.J.); kaaaphapi@163.com (Z.G.); zhangran@163.com (R.Z.); zhuchengsu@zafu.edu.cn (Z.S.); 2 Hangzhou Tea Research Institute, CHINA COOP, Hangzhou 310016, China; sytoneliu@163.com

**Keywords:** NMR, Longjing tea, protected designation of origin, machine learning

## Abstract

Premium green tea is a high-value agricultural product significantly influenced by its geographical origin, making it susceptible to food fraud. This study utilized nuclear magnetic resonance (NMR) spectroscopy to perform chemical fingerprint analysis on 78 Longjing tea (LJT) samples from both protected designation of origin (PDO) regions (Zhejiang) and non-PDO regions (Sichuan, Guangxi, and Guizhou) in China. Unsupervised algorithms and heatmaps were employed for the visual analysis of the data from PDO and non-PDO teas while exploring the feasibility of linear and nonlinear machine-learning algorithms in discriminating the origin of LJT. The findings revealed that the nonlinear model random forest (92.2%), exhibited superior performance compared to the linear model linear discriminant analysis (85.6%). The random forest model identified 15 key marker metabolites for the geographical origin of LJT, such as kaempferol glycoside, glutamine, and ECG. The results support the conclusion that the integration of NMR with machine-learning classification serves as an effective tool for the quality assessment and origin identification of LJT.

## 1. Introduction

Longjing tea is a famous Chinese premium green tea, originating from three regions in Zhejiang Province [1]. According to Chinese national standard (GB/T 18650-2008) [2], flat green tea produced outside the Xihu area and Qiantang area in Hangzhou City, as well as the Yuezhou area in Shaoxing City, Zhejiang Province, cannot be marketed under the label “Longjing Tea” [3]. However, unscrupulous traders often mislabel green tea from other regions as LJT to deceive consumers and gain higher profits [4]. Since consumers are willing to pay a premium for LJT with a protected designation of origin (PDO), this leads to fraudulent behavior in the tea market [5]. Therefore, the development of identification techniques for the origin of LJT is of great significance for the protection of consumers’ rights and interests as well as for the quality supervision of the market sector.

Traditional tea origin identification differentiates tea based on attributes such as appearance, aroma, taste, and tea color [6,7]. However, these sensory reviewers require long-term training, are subjective in their conclusions, and are susceptible to environmental factors. Therefore, researchers are keen to develop objective tea quality assessment methods to replace the traditional sensory review. Over the past decade, various methods have been proposed to determine the geographical origin of tea. These methods include analyzing the chemical composition, elemental composition, and spectral fingerprints of tea, as well as by using combinations of these approaches. For instance, Ma et al. employed inductively coupled plasma mass spectrometry (ICP-MS) to differentiate Biluochun green tea samples from three distinct regions [8]. Although the discrimination rate reached 96.4%, the method requires a complex pre-treatment process that consumes significant sample preparation time, making it impractical for constructing the large datasets needed for origin traceability. On the other hand, Yun et al. used head-space gas chromatography-mass spectrometry (HS-GC/MS) to achieve a 100% origin identification rate for several black teas [9]. However, mass spectrometry analyses usually take a long time and depend on the experience of the spectrometry. Many researchers have also tried to differentiate tea origins by analytical methods such as high-performance liquid chromatography (HPLC) [10] and stable isotope ratio mass spectrometry (IRMS) [11]. However, these methods are still inefficient and there is an urgent need to develop faster methods for origin tracing.

NMR spectroscopy is a rapid technique for sample preparation and data acquisition, offering the advantage of minimal sample processing and consistent data generation. This makes it applicable to various purposes in determining the origin of food products [12]. For example, Cui et al. achieved a 95.7% origin discrimination rate for four Huajiao origins using ^1^H NMR combined with chemometrics [13]. Recently, Cui et al. also achieved a 92.7% discrimination rate for 219 black tea samples from seven origins using ^1^H NMR combined with a machine-learning algorithm [14]. By combining the fast data acquisition capability of ^1^H NMR with chemometric analysis methods, it provides a viable solution for tea origin traceability. Widely used analytical and visual chemometrics methods include principal component analysis (PCA) and projection to latent structures discriminant analysis (PLS-DA) [15]. Machinelearning algorithms are increasingly replacing traditional data processing methods due to their potential to improve discriminant performance, minimize the risk of overfitting, and eliminate irrelevant features. These algorithms can be categorized as linear and non-linear. Linear discriminant analysis (LDA) is a commonly used linear approach in machine learning. It assumes that the data in each category is normally distributed and has the same covariance matrix, aiming to find linear combinations of features that best discriminate between multiple categories [16]. However, LDA can only create linear decision boundaries and may not capture the complex relationships in the data. In contrast, random forest (RF) is an ensemble learning method that enhances classification by constructing multiple decision trees during training and outputting the class predictions of each tree [17]. Because RF combines the predictions of multiple trees, it reduces the risk of overfitting and has the ability to handle a wide range of input variables without eliminating any. Moreover, RF can provide feature variables that are more important for discrimination, which helps to understand the key variables that affect the origin of the food. Hence, it is valuable to investigate the efficacy of both linear and nonlinear models in discerning the geographical source of LJT based on metabolite analysis.

This study investigated the application of ^1^H NMR-based methods combined with machine-learning algorithms in LJT origin identification. By analyzing the metabolic fingerprints of 78 samples from four major LJT-producing regions in China (Zhejiang, Guizhou, Sichuan, and Guangxi), linear (LDA) and non-linear (RF) machine-learning models for origin discrimination were developed. In addition, potential chemical markers for distinguishing LJT origin were revealed. The results of the study can be applied to the origin traceability of LJT and provide a new approach for the quality control of LJT.

## 2. Materials and Methods

### 2.1. Longjing Tea Sample Preparation

**Figure 1:**
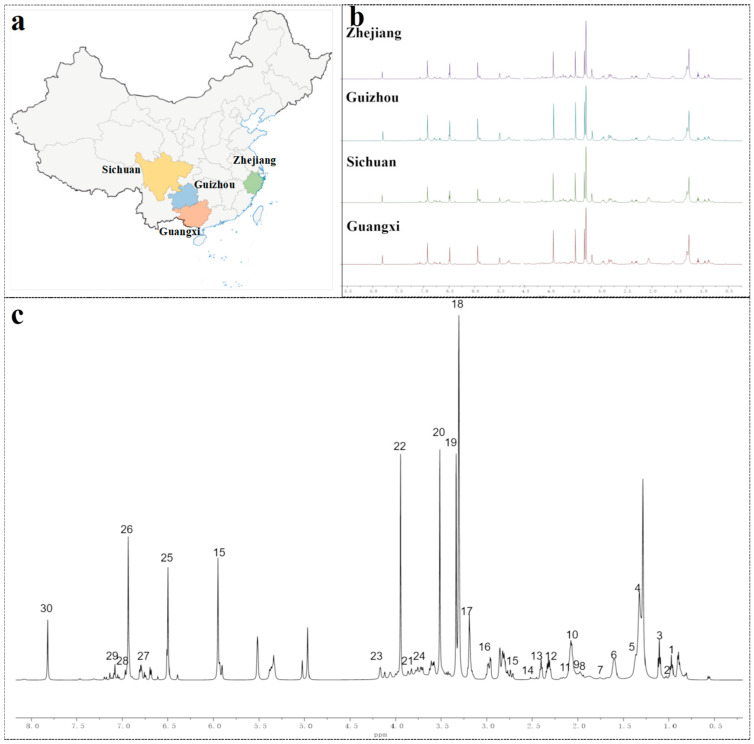
Geographical origin and characteristic ^1^H NMR profiles of LJT samples. (**a**) Samples of LJT were gathered based on their designated geographical source. (**b**) Comparative ^1^H NMR spectra of LJT extracts sourced from diverse geographical regions. (**c**) ^1^H NMR spectrum of LJT depicted in a representative manner.

A total of 78 Longjing tea samples were collected from reliable suppliers (Figure 1a). These samples originated from various regions in China, including Zhejiang (42), Guizhou (12), Sichuan (9), and Guangxi (15). The samples were processed from the raw materials of three varieties of *Camellia sinensis* (Quntizhong, Longjing 43, and Wuniuzao), and their detailed information is shown in Table S1. The authenticity of the samples was confirmed by our collaborators. After the collection of samples, they were transferred to the laboratory in vacuum-sealed packages.

The extraction procedure follows the methodology outlined by Cui et al. [14]. Initially, all tea samples underwent grinding for a duration of 30 s utilizing an IKA A11basic grinder (manufactured in Germany). Following this, the samples were sifted through a 3 mm mesh sieve. Subsequently, 200 mg of each processed sample was blended with 3 mL of methanol-d4, which contained 0.03% Tetramethylsilane (TMS), and subjected to ultrasonic extraction at 600 W for 10 min. This was followed by centrifugation for 5 min at 15,000× *g*. Next, 600 μL of the supernatant was carefully collected and transferred into an NMR tube with a diameter of 5 mm. Each tea sample was prepared three times, then measured, and the average was calculated.

### 2.2. NMR Spectroscopy Detection

NMR detection was conducted in accordance with established methods [14]. All spectra were recorded using a 600 MHz NMR spectrometer (Bruker BioSpin GmbH, Rheinstetten, Germany) equipped with an ultra-low temperature probe. We employed a standard Bruker pulse sequence, with a spectral width spanning from −2 to 14 ppm, a center frequency set at 3600 Hz, and a test temperature maintained at 298 K. The observations were conducted at a frequency of 600 MHz, utilizing a pulse width of 10.25 μs. Each spectrum was acquired over a duration of 4.00 s, with a delay of 1 s between scans, and a total of 31 scans were performed. Corrections for spectral shifts were made based on the TMS signal (*δ* = 0 ppm) in the ^1^H NMR spectra. Prior to Fourier transformation, an exponential plus weighting function, corresponding to a linewidth of 0.3 Hz, was implemented.

Regions corresponding to methanol (3.31–3.34 ppm) and TMS (0 ppm) were excluded from the analysis. Signal assignments were verified by comparing with literature sources [13] and cross-referenced using the Human Metabolome Database (HMDB; http://www.hmdb.ca/ (accessed on 5 July 2024)).

### 2.3. Data Analysis

Phase and baseline corrections were applied to the ^1^H NMR spectra in the MestReNova software (Version 14.0) using the Whitakker smoothing algorithm, and a displacement calibration was performed based on the TMS internal standard at 0.0 ppm. Data reduction was performed using rectangular bins (0.04 ppm) generated in the MestReNova software, with each bin integrated by summing all intensities within that bin. The bin width of 0.04 ppm represents a compromise between maintaining sufficient data resolution and minimizing the effects of loss of spectral information and peak drift to ensure accurate peak integration. The overall intensity of the spectra was normalized using MestReNova.

PCA and sparse PLS discriminant analysis (sPLS-DA) were performed utilizing the MetaboAnalyst 5.0 online platform (https://www.metaboanalyst.ca (accessed on 5 July 2024)). To mitigate data overfitting and ensure the robustness of supervised analyses, permutation testing (*n* = 2000) and cross-validation techniques were applied. Additionally, heatmap generation and hierarchical clustering (HC) were conducted using MetaboAnalyst 5.0, with inter-group similarities assessed through Pearson distance metrics. The violin chart was drawn according to the relative value of the peak intensity obtained by the spectral division box.

All machine-learning procedures were executed in MATLAB R2021b (Mathworks, Waltham, MA, USA). The tea samples were divided into a training set (52 samples) and a test set (26 samples) with a 2:1 ratio. To enhance algorithm reliability, a 5-fold cross-validation strategy was employed. Linear Discriminant Analysis (LDA) provided optimal separation by projecting high-dimensional data into a discriminant vector space, thereby extracting classification information and reducing dimensionality. Random forest (RF), an ensemble method, aggregates multiple decision trees through bagging, which involves creating numerous subsets and combining several decision trees. Each subset is randomly sampled with replacement, and certain features are randomly selected as inputs, with the final classification result based on the majority vote. In this study, a random forest with 5000 trees was used to achieve superior classification performance. The efficacy of the machine-learning algorithms was evaluated using a confusion matrix.

## 3. Results and Discussion

### 3.1. Metabolomic Analysis of Longjing Tea

In this study, the metabolite composition of 78 LJT samples from four regions was assessed using ^1^H NMR. The ^1^H NMR spectra of LJT samples from Zhejiang, Guizhou, Sichuan, and Guangxi are depicted in Figure 1b. Preliminary comparative analysis revealed that LJT from Zhejiang and Guizhou exhibited heightened peaks in the high-field region (0.8–3.5 ppm, corresponding to amino acids) compared to those originating from Sichuan and Guangxi. In the mid-to-low field region (3.5–5.5 ppm, corresponding to carbohydrates), Zhejiang and Sichuan samples displayed similar peaks. In the low-field region above 6 ppm (aromatic compounds), Guizhou and Guangxi samples had higher peaks than those from Zhejiang and Sichuan. These findings suggest that the compound composition of LJT samples varied in different regions.

A representative ^1^H NMR spectrum of LJT samples can be observed in Figure 1c. Based on previously reported chemical shifts and combined with public metabolomics databases [14,18,19,20], 30 metabolites were identified (Table 1). LJT extracts contain a diverse array of compounds, including tea polyphenols such as epigallocatechin gallate (EGCG), epicatechin (EC), epigallocatechin (EGC), and epicatechin gallate (ECG). Moreover, they also encompass caffeine and amino acids like theanine, isoleucine, and leucine, as well as organic acids including quinic acid, malic acid, and succinic acid. Additionally, these extracts are characterized by the presence of carbohydrates such as *α*-glucose, *β*-glucose, and sucrose. In prior research, it has been observed that the bitter taste and astringency of green tea can be attributed to the existence of EGCG and ECG, which may be influenced by factors like the type and quality of tea leaves. The umami flavor of green tea is primarily attributed to the presence of theanine, which exhibits a strong correlation with the timing of raw material harvest. This suggests that using ^1^H NMR for metabolite fingerprinting analysis can reflect differences in the quality of the raw materials used in LJT from different regions.

### 3.2. PCA and sPLS-DA Analysis of Longjing Tea Origin

**Figure 2:**
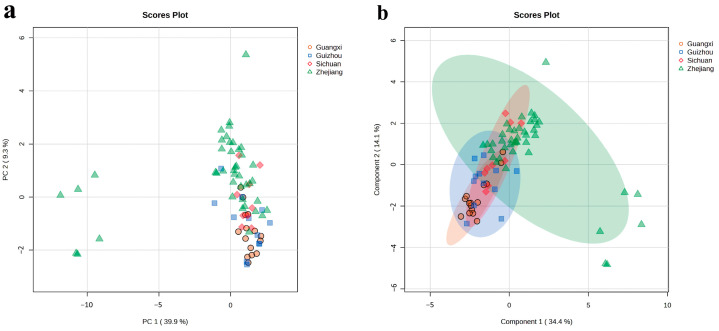
Principal component analysis (PCA) (**a**) and sparse PLS discriminant analysis (sPLS-DA) (**b**) of all 78 LJT samples.

To evaluate the classification accuracy of LJT, PCA was employed using ^1^H NMR chemical fingerprints. Additionally, it helped in visualizing the distinction between different groups and the variability within each group (Figure 2a). Given that principal components (PCs) are formed by linearly combining the original variables, the visualization of PCA is limited in its ability to capture the entirety of variance [21]. The PCA results indicated an overlap among samples in the PCA score plot, with notable similarities between samples from Guangxi and Guizhou, likely due to their geographical proximity. Interestingly, similarities were also observed between samples from Zhejiang and Sichuan, suggesting that geographical factors significantly influence tea quality [22].

To conduct a more in-depth analysis of the variations in metabolites among the four regions where LJT is produced, we utilized a supervised model known as sPLS-DA to compare and contrast the distinct groups. The sPLS-DA results indicated that distinguishing the four production areas remained challenging (Figure 2b). This difficulty was primarily manifested in the high similarity between samples from Sichuan and Zhejiang, with some samples from Guangxi and Guizhou also overlapping with those from Zhejiang. Previous studies have demonstrated that using supervised models for origin identification can be challenging when samples exhibit highly overlapping or similar metabolite characteristics, posing challenges for the model in recognizing an adequate number of sufficient distinguishing features [23]. LJT from different production areas contains similar primary metabolites; although the concentrations of these metabolites may vary, these differences remain insufficient for the sPLS-DA model to accurately distinguish between the production areas.

### 3.3. Hierarchical Clustering of Longjing Tea Origins

**Figure 3:**
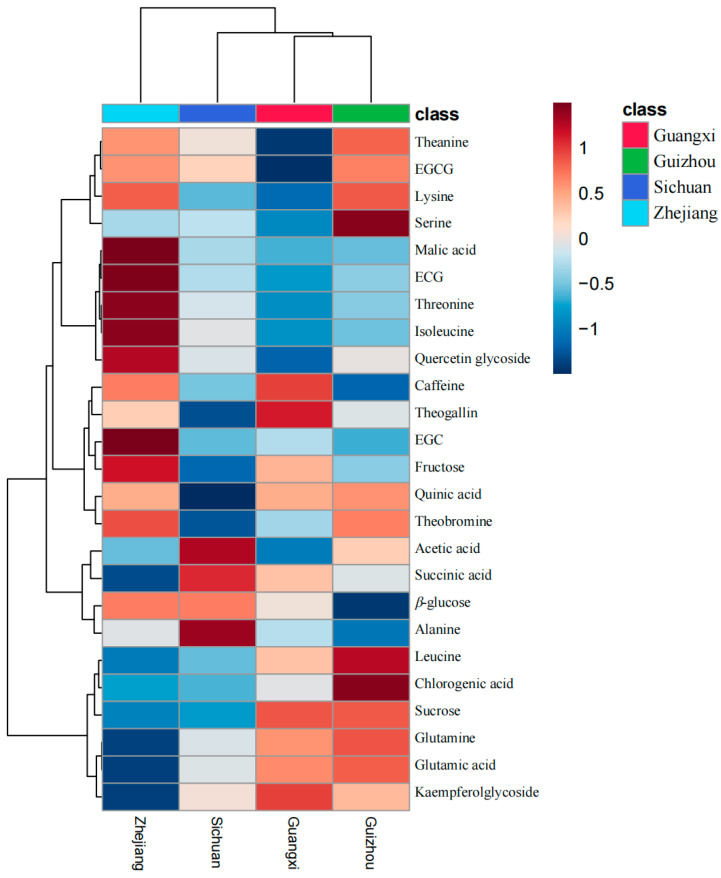
Geographic origin-based hierarchical clustering of LJT using ^1^H NMR spectra.

Heatmap and hierarchical clustering analyses were employed to visualize the metabolites of LJT sourced from various geographical origins (Figure 3). Hierarchical clustering was performed based on the mean values of 25 metabolites selected by ANOVA across samples from four origins. The grouping of LJT samples by origin revealed three hierarchical branches. The first branch includes samples from Guangxi and Guizhou; the second branch comprises samples from Guangxi, Guizhou, and Sichuan; and the third branch indicates that Zhejiang differs from the other three origins. The hierarchical clustering roughly corresponds to the geographical proximity of the origins. LJT from Zhejiang exhibits comparatively elevated levels of polyphenols, amino acids, and alkaloids in comparison to other regions. Conversely, Zhejiang demonstrates relatively diminished concentrations of certain organic acids (such as acetic acid, succinic acid, and chlorogenic acid) and sucrose when compared to the aforementioned regions. The main flavor components of tea are amino acids (umami), flavonoids (bitter and astringent), and alkaloids (bitter) [24]. Therefore, the differences in these substances in the LJT provide clues for origin discrimination and quality assessment.

### 3.4. Machine-Learning Algorithm for Longjing Tea Origins

**Figure 4:**
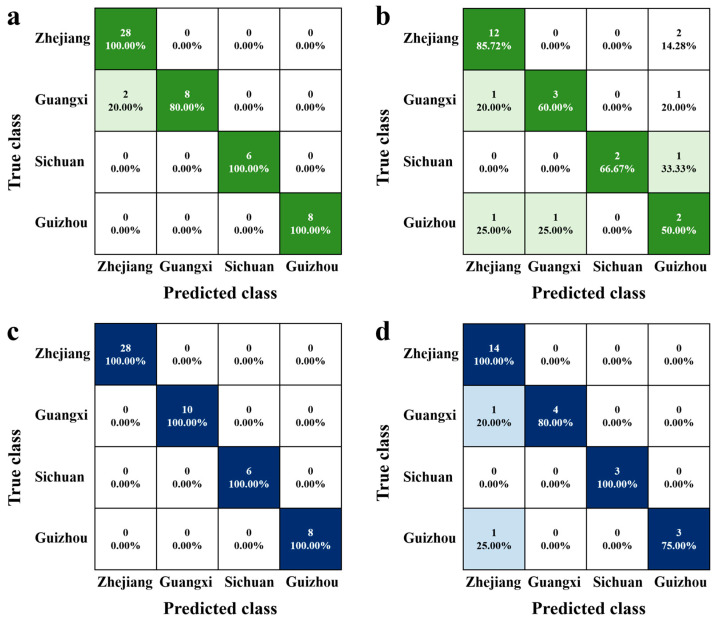
The confusion matrices for different models were obtained using all the data from 78 LJT samples from four production areas. (**a**) Training set of LDA. (**b**) Testing set of LDA. (**c**) Training set of RF. (**d**) Testing set of RF. The darker the green and blue colours in the matrix squares, the higher the accuracy rate.

To enhance the categorization of LJT samples originating from diverse sources, a range of classification algorithms with varying attributes (linear/non-linear) were examined to identify the most suitable method for tackling intricate pattern classification issues. LDA, a well-known linear model, aims to enhance sample discrimination by maximizing inter-class variance while minimizing intra-class variance [25]. The LDA training set achieved a classification accuracy of 96.2% (Figure 4a), while the testing set demonstrated an accuracy rate of 85.6% (Figure 4b). The classification accuracy for Zhejiang reached 85.72%, while the accuracies for Guangxi, Sichuan, and Guizhou were 60%, 66.67%, and 50%, respectively. RF is an innovative ensemble technique employed for machine-learning models, particularly those relying on nonlinear classification trees [26]. The RF algorithm builds numerous classification trees by randomly picking variables (columns) and data instances (rows), subsequently combining the outcomes of these trees for the purpose of classification or regression. The optimized RF model was utilized to distinguish LJT from four distinct sources (Figure 4c,d). In comparison to LDA, RF exhibited superior classification accuracy, achieving a discrimination rate of 100% for the training set and 92.3% for the testing set (Figure 4). Among the samples, those from Guizhou exhibited the highest classification error rate, while Zhejiang and Sichuan samples had the lowest. Specifically, one sample from Guangxi and one from Guizhou were misclassified as Zhejiang. Both of these samples originate from tea plant varieties transplanted from Zhejiang, indicating that the variety of raw material significantly impacts the final tea quality. This finding is consistent with previous research, where Cui et al. reported that the raw material used in tea processing is a major factor affecting origin-related quality differences [14]. The absence of misclassification between Sichuan and Zhejiang LJTs, which have similar dimensions, suggests that climatic conditions are not the primary determinant of tea’s chemical composition. Comparing the prediction results of nonlinear algorithms with those of linear algorithms, it was found that nonlinear algorithms outperform linear algorithms in terms of predictive accuracy [14]. This finding is consistent with previous research, which indicates that metabolite levels in teas from different origins cannot be easily classified using simple linear methods due to the complex interplay of factors such as tea plant varieties, climate, management practices, and processing methods [27]. To enhance the differentiation of quality characteristics among origins, advanced machine-learning algorithms are essential.

### 3.5. Metabolite Biomarkers Differentiating Longjing Tea Origins

**Figure 5:**
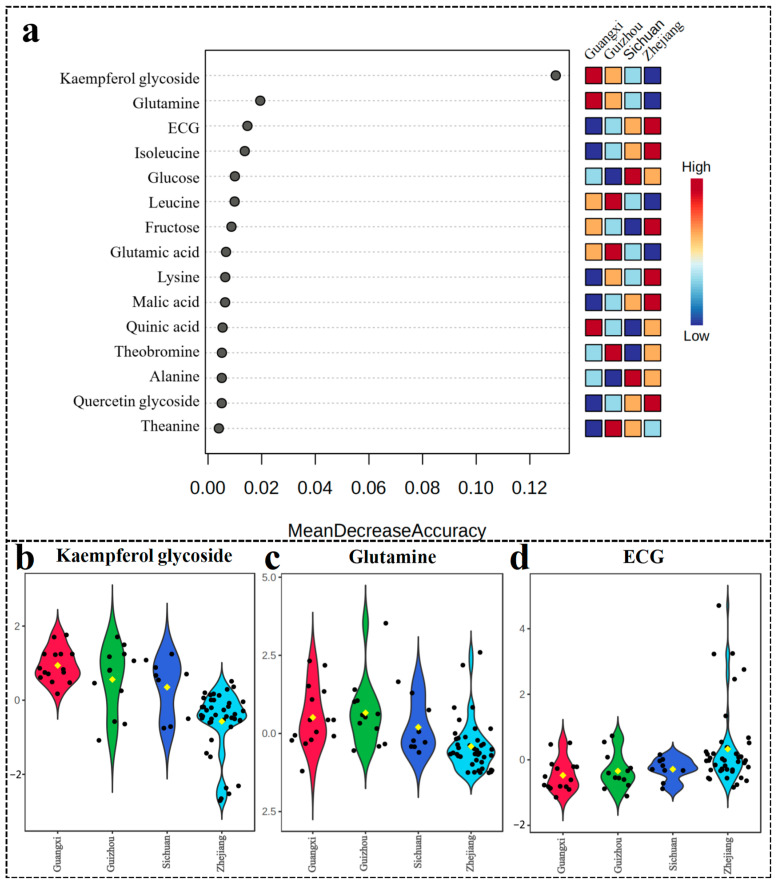
(**a**) Significant features identified by the random forest algorithm; (**b**) Kaempferol glycoside, (**c**) Glutamine, and (**d**) ECG box. The black dots in the fiddle diagram represent the relative content values for each sample, and the yellow dots represent the within-group average values.

Through RF analysis (Figure 5), 15 potential chemical markers distinguishing LJT samples originating from various locales have been meticulously identified. Directly measuring the impact of each feature on the accuracy of the model is what mean decrease accuracy aims to achieve [28]. This is accomplished by rearranging the sequence of feature values and assessing the influence of changes in order on model accuracy. A higher average reduction in accuracy suggests a stronger influence of the variable on RF predictions. In terms of ranking, the metabolite that holds the utmost significance in distinguishing between the four production areas is Kaempferol glycoside. Previous research has indicated that variations in cultivation environments could potentially exert a substantial influence on the structural alterations observed in specific flavonoid metabolites within LJT of identical varieties [29]. It has been proposed that geographical origin has less impact on flavonoid metabolites compared to cultivation variety. Consistent with this, our study found that the content of Kaempferol glycoside is primarily dependent on the genetic characteristics of the tea plant, with less impact from the cultivation variety on quercetin glycoside. Quercetin glycoside and kaempferol glycoside contribute to the mild astringency of the tea brew and enhance the bitterness of caffeine, making them important components of the tea flavor [30]. Our study discovered that LJT from Zhejiang has lower levels of kaempferol glycoside compared to other regions, while quercetin glycoside content is higher than in other regions (Figure 5a). This may be due to the predominant use of specific varietal materials, although the high taste threshold of flavonoid glycosides makes it difficult for assessors or consumers to detect taste differences in the tea infusion. Previous research has utilized glutamine, a prominent umami amino acid found in tea, as a significant indicator for differentiating between various types of green tea [31]. Recent studies have shown that extended processing time and heating temperature reduce glutamine content [32]. Our study found that glutamine levels are lower in Zhejiang samples compared to other regions, possibly due to different processing conditions and temperatures, indicating that processing methods are a significant factor affecting the quality of LJT from different origins.

Furthermore, the RF model’s discrimination results are affected by an additional set of five amino acids (Theanine, Alanine, Lysine, Leucine, and Isoleucine), ranked in descending order of significance. In green tea, isoleucine, leucine, and lysine contribute to bitterness, alanine is considered a sweet amino acid, and theanine is regarded as the primary umami amino acid [33]. The variation in the origin of LJT may result in differences in its taste due to variances in amino acid composition. Previous research has suggested that certain amino acids (proline, valine, and glutamic Acid) exhibit stability throughout the processing stages, whereas others (isoleucine, leucine, lysine, alanine, and theanine) are more prone to reduction during processing [34]. This suggests that differences in amino acids in LJT may be primarily influenced by processing, with these six amino acids helping to differentiate closely related geographic regions. ECG is one of the primary contributors to the bitterness and astringency of tea infusion [35]. The higher content of ECG in LJT from Zhejiang compared to other regions is primarily due to the combined effects of cultivar selection, agronomic practices, and processing methods. Glucose and fructose contribute sweetness to tea infusion. Prior research has suggested a correlation between the temperature at which tea leaves grow and the buildup of carbohydrates in them [36]. Our research found that the glucose and fructose content in tea from Zhejiang and Sichuan is higher compared to that from Guizhou and Guangxi. This disparity may be attributed to the fact that LJT from Guizhou and Guangxi is harvested from tea plants grown in southern China, where the environment features higher light intensity and temperatures, leading to lower carbohydrate accumulation in the tea plants [37]. Conversely, in the core production area of LJT, higher quality standards are typically enforced. As a result, only the new shoots from early spring, just after winter, are collected for processing into LJT. In contrast, in other regions, new tea shoots are collected and processed throughout both spring and summer, during which carbohydrate accumulation in tea leaves is lower, resulting in a reduced sweetness in the tea infusion.

In recent studies, ^1^H NMR has been recognized as an effective method for assessing the origin of black tea [14]. Our study reveals that ^1^H NMR also exhibits considerable potential in assessing the provenance of green tea. Previous investigations have identified glucose, sucrose, EGCG, EGC, EC, caffeine, theanine, alanine, and threonine as crucial indicators for distinguishing the source of green tea [38]. Our study confirms the significance of glucose, caffeine, and theanine in tea origin identification and reveals that, for LJT, key distinguishing compounds include kaempferol glycoside, which is significantly affected by cultivar; glutamine, which is influenced by processing; and ECG, which is impacted by multiple factors. RF modeling indicates that the key variables for differentiating the origin of LJT are predominantly influenced by cultivar and processing methods. The importance of these variables highlights that the varietal variations play a pivotal role in differentiating the source of LJT.

In brief, we have devised a quick and uncomplicated technique for determining the source and assessing the quality of Longjing tea. In contrast to the 40 min digestion period needed for identifying green tea origin through ICP-MS [8] and the 42 min detection time required for black tea origin identification using HS-GC/MS [9], our sample preparation requires only 15 min, followed by a detection time of only 2 min. This significant reduction in processing time facilitates the establishment of large datasets. In addition, the high reproducibility of NMR results compared to analytical methods such as LC-MS [39] and GC-MS [40] enhances the stability of model performance in academic research. Considering the influence of production and storage years on tea metabolites, more LJT samples from different years still need to be collected for analysis in practical applications to help optimize the machine-learning model to ensure the accuracy of the identification results.

## 4. Conclusions

This study employed a combination of ^1^H NMR chemical fingerprints and machine-learning algorithms to analyze 78 Longjing tea samples from four major production regions. In these samples, a total of 30 metabolites were identified, including tea polyphenols, organic acids, carbohydrates, and alkaloids. Accurate identification of the origin of Longjing tea was achieved using ^1^H NMR chemical fingerprint information combined with a nonlinear algorithm (random forest), achieving an identification rate of 92.3%. The study thoroughly discussed the impact of raw material cultivar and processing conditions on the discrimination results. By analyzing the average decrease in random forest classification accuracy, 15 important variables were identified. Kaempferol glycoside, significantly affected by cultivar; glutamine, influenced by processing; and ECG, impacted by both cultivar and processing, were identified as major discriminatory factors. The findings suggest that the utilization of machine-learning algorithms in conjunction with ^1^H NMR can serve as an efficient approach to assess the excellence and source of high-grade green teas, thereby contributing to quality management within the tea industry. This methodology offers a swift, consistent, and replicable means for certifying the origin of various agricultural products or food items.

## Figures and Tables

**Table 1:** Thirty major metabolites were identified through the detection of ^1^H NMR signals in methanol extracts obtained from LJT samples originating from four distinct geographical locations.

No.	Metabolite	Chemical Shift, in ppm (Multiplicity)
1	Leucine	0.97(d)
2	Isoleucine	1.03, 1.98
3	Theanine	1.10, 2.13, 2.37, 3.19, 3.72
4	Threonine	1.36, 4.23
5	Alanine	1.46 (d), 3.84
6	Arginine	1.68 (m), 1.90 (m)
7	Lysine	1.71 (m), 1.87 (m)
8	Glutamine	2.01
9	Quinic acid	2.05 (m), 3.54 (dd), 4.04 (dd)
10	Acetic acid	2.07
11	Glutamic acid	2.12 (m)
12	Chlorogenic acid	2.17 (m), 5.33 (m)
13	Malic acid	2.37 (dd), 2.63 (dd)
14	Succinic acid	2.52 (s)
15	EGC	2.62, 4.27, 6.06, 6.55, 6.80
16	EGCG	2.72, 3.08, 5.56, 6.59, 6.92
17	ECG	3.08, 4.81, 6.50, 6.95
18	Caffeine	3.22 (s), 3.38 (s), 3.77 (s)
19	Sucrose	3.43, 3.65, 3.70, 4.08, 4.23
20	*α*-glucose	3.50, 5.16 (d)
21	Theogallin	2.20, 3.84, 4.20
22	Serine	3.83, 3.97 (m)
23	Fructose	3.56, 4.13
24	*β*-glucose	3.58, 4.58
25	Rutin	4.52 (d), 5.11 (d), 6.39 (d)
26	EC	6.04, 6.11, 6.50, 6.87, 6.99
27	Quercetin glycoside	6.88, 7.63
28	Kaempferol glycoside	6.96
29	Gallic acid	7.07 (s)
30	Theobromine	7.81

## Data Availability

The original contributions presented in the study are included in the article/Supplementary Material, further inquiries can be directed to the corresponding author.

## Supplementary Materials

The following supporting information can be downloaded at: https://www.mdpi.com/article/10.3390/foods13172702/s1, Table S1: Detailed sources of Longjing tea samples.
