# Supplementary material for: 1H NMR Spectroscopy Combined with Machine-Learning Algorithm for Origin Recognition of Chinese Famous Green Tea Longjing Tea
Source: Foods. 2024 Aug 27;13(17):2702. doi: 10.3390/foods13172702 (PMC11394610; doi:10.3390/foods13172702)
Supplement: Supplementary file 1 [file foods-13-02702-s001.zip › foods-3145647-supplementary.pdf]

Table S1. Detailed sources of Longjing tea samples.

Sample Name	Year	Province	Region	Variety
ZJ 1	2023	Zhejiang	Xihu area	Quntizhong
ZJ 2	2023	Zhejiang	Xihu area	Quntizhong
ZJ 3	2023	Zhejiang	Xihu area	Quntizhong
ZJ 4	2023	Zhejiang	Xihu area	Quntizhong
ZJ 5	2023	Zhejiang	Xihu area	Quntizhong
ZJ 6	2023	Zhejiang	Xihu area	Quntizhong
ZJ 7	2023	Zhejiang	Xihu area	Quntizhong
ZJ 8	2023	Zhejiang	Xihu area	Quntizhong
ZJ 9	2023	Zhejiang	Xihu area	Quntizhong
ZJ 10	2023	Zhejiang	Xihu area	Quntizhong
ZJ 11	2023	Zhejiang	Xihu area	Quntizhong
ZJ 12	2023	Zhejiang	Xihu area	Quntizhong
ZJ 13	2023	Zhejiang	Xihu area	Longjing 43
ZJ 14	2023	Zhejiang	Xihu area	Longjing 43
ZJ 15	2023	Zhejiang	Xihu area	Longjing 43
ZJ 16	2023	Zhejiang	Qiantang area	Longjing 43
ZJ 17	2023	Zhejiang	Qiantang area	Longjing 43
ZJ 18	2023	Zhejiang	Qiantang area	Longjing 43
ZJ 19	2023	Zhejiang	Qiantang area	Quntizhong
ZJ 20	2023	Zhejiang	Qiantang area	Quntizhong
ZJ 21	2023	Zhejiang	Qiantang area	Quntizhong
ZJ 22	2023	Zhejiang	Qiantang area	Quntizhong
ZJ 23	2023	Zhejiang	Qiantang area	Quntizhong
ZJ 24	2023	Zhejiang	Qiantang area	Quntizhong
ZJ 25	2023	Zhejiang	Qiantang area	Quntizhong
ZJ 26	2023	Zhejiang	Qiantang area	Quntizhong
ZJ 27	2023	Zhejiang	Qiantang area	Quntizhong
ZJ 28	2023	Zhejiang	Yuezhou area	Longjing 43
ZJ 29	2023	Zhejiang	Yuezhou area	Longjing 43
ZJ 30	2023	Zhejiang	Yuezhou area	Longjing 43
ZJ 31	2023	Zhejiang	Yuezhou area	Quntizhong
ZJ 32	2023	Zhejiang	Yuezhou area	Quntizhong
ZJ 33	2023	Zhejiang	Yuezhou area	Quntizhong
ZJ 34	2023	Zhejiang	Yuezhou area	Quntizhong
ZJ 35	2023	Zhejiang	Yuezhou area	Quntizhong
ZJ 36	2023	Zhejiang	Yuezhou area	Quntizhong
ZJ 37	2023	Zhejiang	Yuezhou area	Quntizhong
ZJ 38	2023	Zhejiang	Yuezhou area	Quntizhong
ZJ 39	2023	Zhejiang	Yuezhou area	Quntizhong
ZJ 40	2023	Zhejiang	Yuezhou area	Quntizhong
ZJ 41	2023	Zhejiang	Yuezhou area	Quntizhong
ZJ 42	2023	Zhejiang	Yuezhou area	Quntizhong
GX 1	2023	Guangxi	Sanjiang County	Longjing 43
GX 2	2023	Guangxi	Sanjiang County	Longjing 43
GX 3	2023	Guangxi	Sanjiang County	Longjing 43
GX 4	2023	Guangxi	Sanjiang County	Wuniuzao

Sample Name	Year	Province	Region	Variety
GX 5	2023	Guangxi	Sanjiang County	Wuniuzao
GX 6	2023	Guangxi	Sanjiang County	Wuniuzao
GX 7	2023	Guangxi	Sanjiang County	Longjing 43
GX 8	2023	Guangxi	Sanjiang County	Longjing 43
GX 9	2023	Guangxi	Sanjiang County	Longjing 43
GX 10	2023	Guangxi	Sanjiang County	Wuniuzao
GX 11	2023	Guangxi	Sanjiang County	Wuniuzao
GX 12	2023	Guangxi	Sanjiang County	Wuniuzao
GX 13	2023	Guangxi	Sanjiang County	Quntizhong
GX 14	2023	Guangxi	Sanjiang County	Quntizhong
GX 15	2023	Guangxi	Sanjiang County	Quntizhong
SC 1	2023	Sichuan	Chengdu City	Quntizhong
SC 2	2023	Sichuan	Chengdu City	Quntizhong
SC 3	2023	Sichuan	Chengdu City	Quntizhong
SC 4	2023	Sichuan	Emei City	Wuniuzao
SC 5	2023	Sichuan	Emei City	Wuniuzao
SC 6	2023	Sichuan	Emei City	Wuniuzao
SC 7	2023	Sichuan	Chengdu City	Quntizhong
SC 8	2023	Sichuan	Chengdu City	Quntizhong
SC 9	2023	Sichuan	Chengdu City	Quntizhong
GZ 1	2023	Guizhou	Xingyi City	Quntizhong
GZ 2	2023	Guizhou	Xingyi City	Quntizhong
GZ 3	2023	Guizhou	Xingyi City	Quntizhong
GZ 4	2023	Guizhou	Kaili City	Quntizhong
GZ 5	2023	Guizhou	Kaili City	Quntizhong
GZ 6	2023	Guizhou	Kaili City	Quntizhong
GZ 7	2023	Guizhou	Liping Country	Longjing 43
GZ 8	2023	Guizhou	Liping Country	Longjing 43
GZ 9	2023	Guizhou	Liping Country	Longjing 43
GZ 10	2023	Guizhou	Kaili City	Quntizhong
GZ 11	2023	Guizhou	Kaili City	Quntizhong
GZ 12	2023	Guizhou	Kaili City	Quntizhong
